# Laplace transform homotopy perturbation method for the approximation of variational problems

**DOI:** 10.1186/s40064-016-1755-y

**Published:** 2016-03-05

**Authors:** U. Filobello-Nino, H. Vazquez-Leal, M. M. Rashidi, H. M. Sedighi, A. Perez-Sesma, M. Sandoval-Hernandez, A. Sarmiento-Reyes, A. D. Contreras-Hernandez, D. Pereyra-Diaz, C. Hoyos-Reyes, V. M. Jimenez-Fernandez, J. Huerta-Chua, F. Castro-Gonzalez, J. R. Laguna-Camacho

**Affiliations:** Facultad de Instrumentación Electrónica, Universidad Veracruzana, Circuito Gonzalo Aguirre Beltrán S/N, 91000 Xalapa, Veracruz Mexico; Shanghai Key Lab of Vehicle Aerodynamics and Vehicle Thermal Management Systems, Tongji University, 4800 Cao An Rd., Jiading, Shanghai, 201804 China; ENN-Tongji Clean Energy Institute of Advanced Studies, Shanghai, China; Department of Mechanical Engineering, Shahid Chamran University, Ahvaz, Iran; Doctorado en Ciencia, Cultura y Tecnología, Universidad de Xalapa, Km 2 Carretera Xalapa-Veracruz, 91190 Xalapa, Veracruz Mexico; National Institute for Astrophysics, Optics and Electronics, Luis Enrique Erro #1, Sta. María Tonantzintla, 72840 Puebla, Mexico; Facultad de Ingeniería Electrónica y Comunicaciones, Universidad Veracruzana, Venustiano Carranza S/N, Col. Revolución, 93390 Poza Rica, Veracruz Mexico; Facultad de Ingeniería Mecánica Eléctrica, Universidad Veracruzana, Venustiano Carranza S/N, Col. Revolución, 93390 Poza Rica, Veracruz Mexico

**Keywords:** Homotopy perturbation method, Nonlinear differential equation, Approximate solutions, Laplace transform, Laplace transform homotopy perturbation method, Variational calculus, Euler equation, 34A34

## Abstract

This article proposes the application of Laplace Transform-Homotopy Perturbation Method and some of its modifications in order to find analytical approximate solutions for the linear and nonlinear differential equations which arise from some variational problems. As case study we will solve four ordinary differential equations, and we will show that the proposed solutions have good accuracy, even we will obtain an exact solution. In the sequel, we will see that the square residual error for the approximate solutions, belongs to the interval [0.001918936920, 0.06334882582], which confirms the accuracy of the proposed methods, taking into account the complexity and difficulty of variational problems.

## Background

The calculus of variations is a powerful branch of the analysis with many applications in both pure and practical mathematics (Lanczos 1986). It has also been found that even, the laws of physics can be expressed in a compact and elegant way through variational principles, as occurs with the Lagrange equations of mechanics, which can be deduced from the variational principle of Hamilton. Unlike elementary calculus problems, which seeks to find the points at which a function reaches its maximum and minimum values, the variational calculus considers the problem of some magnitude, whose values depend on a entire curve, throughout an integral (for instance: surface area or descent time). In fact the aim is to find the curve that extremizes the aforementioned quantity in question.

As will be seen in the next section, the procedure that we will follow consist in finding the differential equation for a function, that leads an integral to take an extreme value.

Although some variational problems were solved by using special methods (like Bernoulli’s solution for the brachistochrone problem (see “Basic idea of variational calculus” section) or so-called isoperimetric problem, proposed and solved by the ancient Greeks), it was Euler who presented the variational calculus as a coherent branch of the analysis by discovering the basic differential equation for an extremization curve.

As discussed in the next section, the Euler equation is a nonlinear equation, quite complicated and impossible to solve it in general, and although some applications lead to specific cases which are soluble, it is clear the importance of searching for methods to find approximate solutions to these equations. In fact, nonlinear problems arise in many phenomena, practical or theoretical. For the same reason, several methods focused to find approximate solutions to nonlinear differential equations, algebraic and transcendental equations and integro-differential equations have been reported, such as those based on based on tanh method (Evans and Raslan 2005), exp-function (Xu 2007; Mahmoudi et al. 2008), Adomian’s decomposition method (ADM) (Adomian and Rach 1985, Adomian 1988; Babolian and Biazar 2002; Rach 2012; Fatoorehchi and Abolghasemi 2012, 2013; Fatoorehchi et al. 2014, 2015a, b, c, d; Fatoorehchi and Abolghasemi 2015e; Hashim 2006), parameter expansion (Zhang and Xu 2007), homotopy perturbation method (HPM) (He 1998, 1999, 2003, 2008; Filobello-Nino et al. 2012, 2013c, 2014a, b, 2015b;Ghaderi 2012; Aminikhah and Hemmatnezhad 2012; Aminikhah 2011, 2012; Vazquez-Leal et al. 2012a, b; Fatoorehchi and Abolghasemi 2011), homotopy analysis method (HAM) (Rashidi et al. 2012a, 2012b; Lakestani and Kazemian 2013), series method (Filobello-Nino et al. 2015a), Lie algebra method (Shang 2012, 2013, 2015; Olver 1993; Casas 1996) and perturbation method (PM) (Filobello-Nino et al. 2013a, b) among many others.

A particularly relevant job, which deals with a combined version of the Laplace transform method (LTM) and Adomian’s decomposition method (ADM) (named the H2LTM), it is described in reference (Fatoorehchi and Abolghasemi 2015e). This work introduced a technique for extending the LTM to solve nonlinear differential equations obtaining analytical and accurate solutions.

The rest of this work is organized as follows. “Basic idea of variational calculus” section presents a basic idea of the main results of variational calculus required for this work. In “Standard HPM” section, we introduce the basic idea of standard HPM method. “LT-HPM and some of its modified versions” section presents an introduction of LT-HPM and some of its modified versions useful for this work. Additionally, “Cases study” section presents four cases study of variational problems. In addition a wide discussion on the results is presented in “Discussion” section. Finally, a brief conclusion is given in “Conclusions” section.

## Basic idea of variational calculus

This paper deals with the problem of finding a function $y = y(x)$ with continues second derivatives (Elsgolts 1983; Levi 1980; Simmons 1983; Zelikin 2005; Boyce 1998), in order to find an approximate solution to the variational problem of extreme an integral of the form

1 $$ I(y) = \int_{{x_{1} }}^{{x_{2} }} {f(x,y,y^{\prime } } )dx, $$

so that its graph join two points, namely $\left( {x_{1} ,y_{1} } \right)$ and $\left( {x_{2} ,y_{2} } \right).$

From variational calculus, we know that if $y(x)$ is a function that satisfies the above requirements, then satisfies the Euler equation.

2 $$ \frac{\text{d}}{\text{dx}}\left( {\frac{\partial f}{{\partial y^{\prime}}}} \right) - \frac{\partial f}{\partial y} = 0,\,\,\,{\text{where}}\,\,\,y^{\prime} = dy/dx. $$

Some interesting cases of the integral (1) are (Simmons 1983; Filobello-Nino et al. 2013d):

- The problem of finding the shortest curve joining two given points, requires to minimize:
  3 $$ \int_{{x_{1} }}^{{x_{2} }} {\sqrt {1 + y^{\prime 2} } } dx. $$
- In the problem of finding the curve that generates the surface of revolution of smallest area when revolves about the axis $x$, we have to minimize:
  4 $$ \int_{{x_{1} }}^{{x_{2} }} {2\pi y\sqrt {1 + y^{\prime 2} } } dx. $$
- The problem of brachistochrone, search for the vertical curve without friction that joins two fixed points, through which a particle slides in the shortest possible time (Connor and Robertson 1997, 1998; Filobello-Nino et al. 2013d). This variational problem for the curve of quickest descent, requires minimize:
  5 $$ \int_{{x_{1} }}^{{x_{2} }} {\frac{{\sqrt {1 + y^{\prime 2} } }}{{\sqrt {2gy} }}} dx $$

When the variable *y* does not appear in the function *f*, Euler’s equation (2) is reduced immediately to the form

6 $$ \frac{d}{dx}\left( {\frac{\partial f}{{\partial y^{\prime}}}} \right) = 0, $$

integrating (6), we see that ${{\partial f}/{\partial y^{\prime}}}$ is conserved in according with differential equation

7 $$ \frac{\partial f}{{\partial y^{\prime}}} = c, $$

where *c* is a constant.

On the other side if *f* is not an explicit function of *x*, then Euler’s equation can be expressed in the form

8 $$ h(x,y,y^{\prime}) = c, $$

where

9 $$ h(x,y,y^{\prime}) = y^{\prime}\frac{\partial f}{{\partial y^{\prime}}} - f . $$

It is customary to refer to the solutions of the Euler equation as stationary functions, and the corresponding value of the integral (1) as stationary value. Besides, the solutions to the above mentioned Euler equation, which are unrestricted by the boundary conditions, are called extremals (Simmons 1983).

## Standard HPM

The standard homotopy perturbation method (HPM) was proposed by Ji Huan He, it was introduced to approach various kinds of nonlinear problems. The HPM is considered as a combination of the classical perturbation technique and the homotopy (whose origin is in the topology), but not restricted to small parameters as occur with traditional perturbation methods (He 1998, 1999).

To figure out how HPM works, consider a general nonlinear differential equation in the form

10 $$ A(u) - f(x) = 0, \quad x \in \varOmega, $$

with the following boundary conditions

11 $$ B(u,\partial u/\partial n) = 0, \quad x \in \varGamma, $$

where *A* is a general differential operator, *B* is a boundary operator, *f*(*x*) a known analytical function and Γ is the domain boundary for Ω. *A* can be divided into two operators *L* and *N*, where *L* is linear and *N* nonlinear; so that (10) can be rewritten as

12 $$ L(u) + N(u) - f(x) = 0 . $$

Generally, a homotopy can be constructed as (He 1998, 1999).

13 $$ H(U,p) = (1 - p)[L(U) - L(u_{0} )] + p[L(U) + N(U) - f(x)] = 0, \quad p \in [0,1], \quad x \in \varOmega $$

or

14 $$ H(U,p) = L(U) - L(u_{0} ) + p[L(u_{0} ) + N(U) - f(x)] = 0, \quad p \in [0,1], \quad x \in \varOmega $$

where *p* is a homotopy parameter, whose values are within range of 0 and 1, $u_{0}$ is the first approximation for the solution of (12) that satisfies the boundary conditions.

Assuming that solution for (13) or (14) can be written as a power series of $p$ as

15 $$ U = v_{0} + v_{1} p + v_{2} p^{2} + \cdots. $$

Substituting (15) into (14) and equating identical powers of $$ p $$ terms, there can be found values for the sequence $$ \nu_{0} , $$$$ \nu_{1} , $$$$ \nu_{2} , $$ …

When $p \to 1$, it yields the approximate solution for (1) in the form

16 $$ U = v_{0} + v_{1} + v_{2} + v_{3} \cdots. $$

## LT-HPM and some of its modified versions

### Basic idea of the LT-HPM method

LT-HPM follows the same steps of standard HPM until (14), next we apply Laplace transform on both sides of homotopy equation (14), to obtain (Aminikhah and Hemmatnezhad 2012; Aminikhah 2011, 2012; Filobello-Nino et al. 2013c, 2014a).

17 $$ \Im \left\{ {L(U) - L(u_{0} ) + p[L(u_{0} ) + N(U) - f(r)]} \right\} = 0, $$

(employing the freedom of homotopy formulation, it is possible that LT-HPM substitutes $L(u_{0} )$ for an arbitrary function $z(x)$ provided with some unknown parameters $A,B,C,\ldots$ to be adequately determined [see cases study 3 and 4)].

Using the differential property (170) of LT, we have (Spiegel 1998):

18 $$ s^{n} \Im \left\{ U \right\} - s^{n - 1} U(0) - s^{n - 2} U^{\prime}(0) - \cdots - U^{(n - 1)} (0) = \Im \left\{ {L(u_{0} ) - pL(u_{0} ) + p\left[ { - N(U) + f(r)} \right]} \right\} $$

or

19 $$ \Im (U) = \left( {\frac{1}{{s^{n} }}} \right)\left\{ {s^{n - 1} U(0) + s^{n - 2} U^{\prime}(0) + \cdots+ U^{(n - 1)} (0)} \right\} + \left( {\frac{1}{{s^{n} }}} \right)\Im \left\{ {L(u_{0} ) - pL(u_{0} ) + p\left[ { - N(U) + f(r)} \right]} \right\}, $$

applying inverse Laplace transform to both sides of (19), we obtain

20 $$ U = \Im^{ - 1} \left\{ {\left( {\frac{1}{{s^{n} }}} \right)\left\{ {s^{n - 1} U(0) + s^{n - 2} U^{\prime}(0) + \cdots + U^{(n - 1)} (0)} \right\} + \left( {\frac{1}{{s^{n} }}} \right)\Im \left\{ {L(u_{0} ) - pL(u_{0} ) + p\left[ { - N(U) + f(r)} \right]} \right\}} \right\}. $$

Assuming that the solutions of (12) can be expressed as a power series of $p$

21 $$ U = \sum\limits_{n = 0}^{\infty } {p^{n} } v_{n}. $$

Then substituting (21) into (20), we get

22 $$ \sum\limits_{n = 0}^{\infty } {p^{n} } \nu_{n} = \Im^{ - 1} \left\{ \begin{aligned}& \left( {\frac{1}{{s^{n} }}} \right)\left\{ {s^{n - 1} U(0) + s^{n - 2} U^{\prime}(0) + \cdots + U^{(n - 1)} (0)} \right\} \hfill \\ &\quad + \left( {\frac{1}{{s^{n} }}} \right)\Im \left\{ {L(u_{0} ) - pL(u_{0} ) + p\left[ { - N( \, \sum\limits_{\text{n = 0}}^{\infty } {p^{n} \nu_{n} } ) + f(r)} \right]} \right\} \hfill \\ \end{aligned} \right\}, $$

comparing coefficients of $p$, with the same power leads to

23 $$ \begin{aligned}p^{0} : \nu_{0} &= \Im^{ - 1} \left\{ {\left( {\frac{1}{{s^{n} }}} \right)\left( {s^{n - 1} U(0) + s^{n - 2} U^{\prime}(0) + \cdots + U^{(n - 1)} (0)) + \Im \left\{ {L(u_{0} )} \right\}} \right)} \right\},\\ p^{1} :\nu_{1} &= \Im^{ - 1} \left\{ {\left( {\frac{1}{{s^{n} }}} \right)\left( {\Im \left\{ { - N_{0} (\nu_{0} ) - L\left( {u_{0} } \right) + f(r)} \right\}} \right)} \right\}, \\ p^{2} :\nu_{2} &= \Im^{ - 1} \left\{ {\left( {\frac{1}{{s^{n} }}} \right)\Im \left\{ { - N_{1} (\nu_{0} ,\nu_{1} )} \right\}} \right\}, \\ p^{3} :\nu_{3} &= \Im^{ - 1} \left\{ {\left( {\frac{1}{{s^{n} }}} \right)\Im \left\{ { - N_{2} (\nu_{0} ,\nu_{1} ,\nu_{2} )} \right\}} \right\} ,\\ &\quad \ldots ,\\ p^{j} :\nu_{j} &= \Im^{ - 1} \left\{ {\left( {\frac{1}{{s^{n} }}} \right)\Im \left\{ { - N_{j - 1} (\nu_{0} ,\nu_{1} ,\nu_{2} ,\ldots,\nu_{j - 1} )} \right\}} \right\} , \\ &\quad \ldots.\end{aligned} $$

Assuming that $U(0) = u_{0} = \alpha_{0} , U^{\prime}(0) = \alpha_{1} ,\ldots,U^{n - 1} (0) = \alpha_{n - 1};$ the exact solution may be obtained as follows

24 $$ u = \mathop {\lim }\limits_{p \to 1} U = \nu_{0} + \nu_{1} + \nu_{2} + \cdots. $$

The above notation has emphasized the fact that the nonlinear operator $N$ can be expanded in formal series with respect to the embedding parameter $p$. The same criterion is used throughout “LT-HPM and some of its modified versions” section (Marinca and Herisanu 2011).

### Basic idea of non-linearities distribution Laplace transform-homotopy perturbation method (NDLT-HPM)

Vazquez-Leal et al. (2012a) introduced a modified version of homotopy perturbation method, the nonlinearities distribution homotopy perturbation method (NDHPM), which sometimes eases the solutions searching process for (12) and reduces the complexity of solving differential equations in terms of power series.

As first step, we introduced the following homotopy (Vazquez-Leal et al. 2012a).

25 $$ H(U,p) = (1 - p)[L(U) - L(u_{0} )] + p[L(U) + N(U,p) - f(x,p)] = 0 $$

or

26 $$ H(U,p) = L(U) - L(u_{0} ) + p[L(u_{0} ) + N(U,p) - f(x,p)] = 0 , \quad p \in [0,1]\quad x \in \varOmega $$

It can be noticed that the homotopy function (25) is essentially the same as (13), except for the nonlinear operator $N$ and the non homogeneous function $f$, which contain embedded the homotopy parameter $p$. Thus, a way to introducenon-linearities distribution Laplace transform-homotopy perturbation method (NDLT-HPM) is apply directly, Laplace transform on both sides of homotopy equation (36), to obtain (Filobello-Nino et al. 2014b).

27 $$ \Im \left\{ {L(U) - L(u_{0} ) + p[L(u_{0} ) + N(U,p) - f(x,p)} \right\} = 0 , $$

(more generally, one could substitute $f(x,p)$ in (25) by another function $g(x,p)$ such that, $g(x,p) \to f(x)$ when $p \to 1,$ see cases study above).

Using the differential property of LT, we have (Spiegel 1998).

28 $$ s^{n} \Im \left\{ U \right\} - s^{n - 1} U(0) - s^{n - 2} U^{\prime}(0) - \cdots - U^{(n - 1)} (0) = \Im \left\{ {L(u_{0} ) - pL(u_{0} ) + p\left[ { - N(U,p) + f(x,p)} \right]} \right\} , $$

or

29 $$ \Im (U) = \left( {\frac{1}{{s^{n} }}} \right)\left\{ {s^{n - 1} U(0) + s^{n - 2} U^{\prime}(0) + \cdots+ U^{(n - 1)} (0) + \Im \left\{ {L(u_{0} ) - pL(u_{0} ) + p\left[ { - N(U,p) + f(x,p)} \right]} \right\}} \right\}, $$

applying inverse Laplace transform to both sides of (29), we obtain

30 $$ U = \Im^{ - 1} \left\{ {\left( {\frac{1}{{s^{n} }}} \right)\left\{ {s^{n - 1} U(0) + s^{n - 2} U^{\prime}(0) + \cdots+ U^{(n - 1)} (0) + \Im \left\{ {L(u_{0} ) - pL(u_{0} ) + p\left[ { - N(U,p) + f(x,p)} \right]} \right\}} \right\}} \right\}. $$

Assuming that the solutions of (12) and $f(x,p)$ can be expressed as a power series of $p$

31 $$ U = \sum\limits_{n = 0}^{\infty } {p^{n} } v_{n} (x) , $$

and

32 $$ f = \sum\limits_{m = 0}^{\infty } {p^{m} } f_{m} (x), $$

respectively, where $f_{m} (x)$ are usually obtained by applying Taylor series method to $f(x,p)$.

Then substituting (32) and (31) into (30), we get

33 $$ \sum\limits_{n = 0}^{\infty } {p^{n} } \nu_{n} = \Im^{ - 1} \left\{ \begin{aligned} &\left( {\frac{1}{{s^{n} }}} \right)\left\{ {s^{n - 1} U(0) + s^{n - 2} U^{\prime}(0) + \cdots + U^{(n - 1)} (0)} \right\} \hfill \\ &\quad + \left( {\frac{1}{{s^{n} }}} \right)\Im \left\{ {L(u_{0} ) - pL(u_{0} ) + p\left[ { - N\left( \sum\limits_{\text{n = 0}}^{\infty } {p^{n} \nu_{n} ,p} \right) + \sum\limits_{\text{m = 0}}^{\infty } {p^{m} f_{m} (x)} } \right]} \right\} \hfill \\ \end{aligned} \right\}, $$

comparing coefficients of $p$, with the same power leads to

34 $$ \begin{aligned}p^{0}: \nu_{0}& = \Im^{ - 1} \left\{ {\left( {\frac{1}{{s^{n} }}} \right)\left( {s^{n - 1} U(0) + s^{n - 2} U^{\prime}(0) + \cdots+ U^{(n - 1)} (0)) + \Im \left\{ {L(u_{0} )} \right\}} \right)} \right\}, \\ p^{1} :\nu_{1}& = \Im^{ - 1} \left\{ {\left( {\frac{1}{{s^{n} }}} \right)\left( {\Im \left\{ {N_{0} (\nu_{0} ) - L\left( {u_{0} } \right) + f_{0} (x)} \right\}} \right)} \right\} ,\\ p^{2} :\nu_{2} &= \Im^{ - 1} \left\{ {\left( {\frac{1}{{s^{n} }}} \right)\Im \left\{ {N_{1} (\nu_{0} ,\nu_{1} ,f_{0} ,f_{1} )} \right\}} \right\} ,\\ p^{3} :\nu_{3} &= \Im^{ - 1} \left\{ {\left( {\frac{1}{{s^{n} }}} \right)\Im \left\{ {N_{2} (\nu_{0} ,\nu_{1} ,\nu_{2} ,f_{0} ,f_{1} ,f_{2} )} \right\}} \right\} , \\ &\quad \ldots\\ p^{j} :\nu_{j} &= \Im^{ - 1} \left\{ {\left( {\frac{1}{{s^{n} }}} \right)\Im \left\{ {N_{j - 1} (\nu_{0} ,\nu_{1} ,\nu_{2} ,\ldots,\nu_{j - 1} ,f_{0} ,f_{1} ,f_{2} ,\ldots f_{j - 1} )} \right\}} \right\} \\ &\quad \ldots .\end{aligned} $$

Assuming that the initial approximation has the form: $U(0) = u_{0} = \alpha_{0} , \,\,U^{\prime}(0) = \alpha_{1} ,\ldots,U^{n - 1} (0) = \alpha_{n - 1} ;$ therefore the exact solution may be obtained as follows

35 $$ u = \mathop {\lim }\limits_{p \to 1} U = \nu_{0} + \nu_{1} + \nu_{2} + \cdots $$

### Basic idea of Laplace transform homotopy perturbation method with variable coefficients (CVLT-HPM)

To obtain (18), we assumed that the coefficient of $L(U)$ is one. In this section we will assume the mentioned coefficient is a positive whole power of $x$, thus we rewrite explicitly (17) for this case as (Filobello-Nino et al. 2015b).

36 $$ \Im \left\{ {\left( {x^{m} L(U) - x^{m} z(x)} \right) + p[x^{m} z(x) + N(U) - f(x)]} \right\} = 0 , $$

where $m$ is a positive integer, and employing the versatility and freedom of HPM we have substituted $L(u_{0} )$ for an arbitrary function $z(x)$. We will choose as a trial function $z(x)$, a polynomial provided with some unknown parameters $A,B,C,\ldots$ to be determined [see (44)].

Using the properties (170) and (171), we have (Spiegel 1998).

37 $$ ( - 1)^{m} \frac{{d^{m} \left[ {s^{n} \Im \left\{ U \right\} - s^{n - 1} U(0) - s^{n - 2} U^{\prime}(0) - \cdots - U^{(n - 1)} (0)} \right]}}{{ds^{m} }} = \Im \left\{ {x^{m} z(x) + p\left[ { - z(x)x^{m} - N(U) + f(x)} \right]} \right\}, $$

after integrating *m* times, we obtain

38 $$ \begin{aligned} &s^{n} \Im \left\{ U \right\} - s^{n - 1} U(0) - s^{n - 2} U^{\prime}(0) - \cdots - U^{(n - 1)} (0)\\ &\quad = ( - 1)^{m} \int {\int { \cdot \cdot \int {\Im \left\{ {x^{m} z(x) + p\left[ { - z(x)x^{m} - N(U) + f(x)} \right]} \right\}} } } dsds^{\prime} \cdot\cdot ds^{\prime\prime} \\ &\qquad (m\hbox{-times}), \end{aligned} $$

or

39 $$ U = \Im^{. - 1} \left\{ {\frac{1}{{s^{n} }}\left\{ {s^{n - 1} U(0) + s^{n - 2} U^{\prime}(0) + \cdots + U^{(n - 1)} (0) + ( - 1)^{m} \int {\int { \cdot \cdot \int {\Im \left\{ {r^{m} z(x) + p\left[ { - z(x)x^{m} - N(U) + f(x)} \right]} \right\}} } } dsds^{\prime} \cdot \cdot ds^{\prime\prime}} \right\}} \right\}. $$

Assuming also, that the solutions of the ODE to solve, can be expressed as a power series of $p$

40 $$ U = \sum\limits_{n = 0}^{\infty } {p^{n} } v_{n} . $$

Then substituting (40) into (39), we get

41 $$ \sum\limits_{n = 0}^{\infty } {p^{n} } \nu_{n} = \Im^{ - 1} \left\{ \begin{aligned}& \left( {\frac{1}{{s^{n} }}} \right)\left\{ {s^{n - 1} U(0) + s^{n - 2} U^{\prime}(0) + \cdots+ U^{(n - 1)} (0)} \right\} \hfill \\ &\quad + \left( {\frac{{( - 1)^{m} }}{{s^{n} }}} \right)\int {\int { \cdot \cdot \int {\Im \left\{ {x^{m} z(x) + p\left[ { - x^{m} z(x) - N( \, \sum\limits_{n = 0}^{\infty } {p^{n} \nu_{n} } ) + f(x)} \right]} \right\}} } } dsds^{\prime} \cdot \cdot ds^{\prime\prime} \hfill \\ \end{aligned} \right\}, $$

comparing coefficients of $$ p, $$ with the same power leads to

42 $$ \begin{aligned} p^{0} : \nu_{0} & = \Im^{ - 1} \left\{ {\left( {\frac{1}{{s^{n} }}} \right)\left( {s^{n - 1} U(0) + s^{n - 2} U^{\prime}(0) + \cdots+ U^{(n - 1)} (0)) + \left( { - 1} \right)^{m} \int {\int { \cdot \cdot \int {\Im \left\{ {x^{m} z(x)} \right\}dsds^{\prime} \cdot \cdot ds^{\prime\prime}} } } } \right)} \right\}\\ p^{1} :\nu_{1} & = \Im^{ - 1} \left\{ {\left( {\frac{1}{{s^{n} }}} \right)\left( { - 1} \right)^{m} \int {\int { \cdot \cdot \int {\left( {\Im \left\{ { - N_{0} (\nu_{0} ) - x^{m} z\left( x \right) + f(x)} \right\}} \right)dsds^{\prime} \cdot \cdot ds^{\prime\prime}} } } } \right\}\\ p^{2} :\nu_{2} & = \Im^{ - 1} \left\{ {\left( {\frac{1}{{s^{n} }}} \right)\left( { - 1} \right)^{m} \int {\int { \cdot \cdot \int {\left( {\Im \left\{ { - N_{1} (\nu_{0} ,\nu_{1} )} \right\}} \right)dsds^{\prime} \cdot \cdot ds^{\prime\prime}} } } } \right\}\\ p^{3} :\nu_{3} & = \Im^{ - 1} \left\{ {\left( {\frac{1}{{s^{n} }}} \right)\left( { - 1} \right)^{m} \int {\int { \cdot \cdot \int {\left( {\Im \left\{ { - N_{2} (\nu_{0} ,\nu_{1} ,\nu_{2} )} \right\}} \right)dsds^{\prime} \cdot \cdot ds^{\prime\prime}} } } } \right\} \\ & \quad\ldots, \\ p^{j} :\nu_{j} & = \Im^{ - 1} \left\{ {\left( {\frac{1}{{s^{n} }}} \right)\left( { - 1} \right)^{m} \int {\int { \cdot \cdot \int {\left( {\Im \left\{ { - N_{j - 1} (\nu_{0} ,\nu_{1} ,\nu_{2} ,\ldots\nu_{j - 1} )} \right\}} \right)dsds^{\prime} \cdot \cdot ds^{\prime\prime}} } } } \right\} , \\ &\quad\ldots.\end{aligned} $$

Assuming that $U(0) = u_{0} = \alpha_{0} , U^{\prime}(0) = \alpha_{1} ,\ldots,U^{n - 1} (0) = \alpha_{n - 1} ;$ then an approximate solution may be obtained as follows

43 $$ u = \mathop {\lim }\limits_{p \to 1} U = \nu_{0} + \nu_{1} + \nu_{2} + \cdots. $$

For boundary value problems it is expected that some of the initial conditions above mentioned, be initially unknown; therefore (43) can be expressed as

44 $$ u = u(x,A,B,C,\ldots,\alpha_{i} ). $$

In order to calculate adequately the values for $A,B,C,\ldots,\alpha_{i},$ we will deduce an algebraic system for them, in the following way.

1. Requiring that (44) satisfies the boundary conditions at the endpoint of the interval.
2. In order to find the values of the total number of parameters, we require add more algebraic equations to those found in (1), up to get as many equations as parameters to determine. If are required, say *j* additional equations, then a convenient possibility is incorporating the following *j*-equations (Marinca and Herisanu 2011).
  $$ R\left( {x_{1} ,A,B,C,\ldots,\alpha_{i} } \right) = R\left( {x_{2} ,A,B,C,\ldots,\alpha_{i} } \right) = \cdots = R\left( {x_{j} ,A,B,C,\ldots,\alpha_{i} } \right) = 0 , $$
  where the residual is defined, by substituting of (44) into (12), to obtain(see 176).

$R\left( {x,A,B,C,\ldots,\alpha_{i} } \right) = L( u({x,A,B,C,\ldots,\alpha_{i} })) + N(u({x,A,B,C,\ldots,\alpha_{i} } )) - f(x)$. The above points $x_{1} ,x_{2} ,x_{3} ,\ldots,x_{j}$ belong to the interest interval, and it is assumed that $u(x,A,B,C,\ldots,\alpha_{i} )$ is the approximate solution of (12) given by (33).

For the case of second order ODES, where $y^{\prime}(0) = A \ne 0$; it is possible to show the term containing $y^{\prime}(x)$ could give rise to an inappropriate term $\Im^{ - 1} \left\{ {\frac{Ln(s)}{{s^{2} }}} \right\}.$ To avoid this potential problem, we should perform, as a work rule, an adequate transformation in order to express our differential equation in its normal form (Simmons 1983). Such as it is known, the normal form does not contain the problematic term $y^{\prime}(x)$, and therefore allows applying VCLT-HPM algorithm in accordance with the above procedure (Filobello-Nino et al. 2015b).

## Cases study

This section, applies LT-HPM and some of their modifications aforementioned, in order to find analytical approximate expressions for the stationary functions, solutions of the Euler equations, which turn out from the following cases study.

### Case 1

This case study uses basic version of LT-HPM.

We will get an approximate solution for the problem of finding a function $y(x)$ that extremizes the value of the integral.

45 $$ \int_{{x_{1} }}^{{x_{2} }} {\left( {\frac{1}{2}y^{\prime 2} - y^{3} - x^{2} y^{2} } \right)} dx . $$

After employing Euler equation (2), for $f = \frac{1}{2}y^{\prime 2} - y^{3} - x^{2} y^{2}$ we obtain

$$ y^{\prime\prime}(x) + 3y^{2} (x) + 2x^{2} y(x) = 0 . $$

As a case study, we consider the following boundary conditions $y(0) = 0$,$y(1) = 1/2$ so that we can express formally our problem in the following way

46 $$ y^{\prime\prime}(x) + 3y^{2} (x) + 2x^{2} y(x) = 0, \quad y(0) = 0, \,\, y(1) = 1/2 $$

Unfortunately there is not a systematic way to obtain an exact solution for this boundary value problem.

To obtain an approximate solution for (46), we identify

47 $$ L(y) = y^{\prime\prime}(x) , $$

48 $$ N(y) = 3y^{2} + 2x^{2} y(x) . $$

We construct the following homotopy in accordance with (13)

49 $$ (1 - p)(y^{\prime\prime} - y^{\prime\prime}_{0} ) + p\left[ {y^{\prime\prime} + 3y^{2} + 2x^{2} y} \right] = 0 , $$

or

50 $$ y^{\prime\prime} = y^{\prime\prime}_{0} + p\left[ { - y^{\prime\prime}_{0} - 3y^{2} - 2x^{2} y} \right]. $$

Applying Laplace transform to (50) we get

$$ \Im \left\{ {y^{\prime\prime}} \right\} = \Im \left\{ {y^{\prime\prime}_{0} + p\left[ { - y^{\prime\prime}_{0} - 3y^{2} - 2x^{2} y} \right]} \right\}. $$

As it is explained in (Spiegel 1998), it is possible to rewrite the above equation, as

51 $$ s^{2} Y(s) - sy(0) - y^{\prime}(0) = \Im \left( {y^{\prime\prime}_{0} + p\left( { - y^{\prime\prime}_{0} - 3y^{2} - 2x^{2} y} \right)} \right) , $$

where we have defined $Y(s) = \Im (y(x))$.

After applying the initial condition $y(0) = 0$, the last expression can be simplified as follows

52 $$ s^{2} Y(s) - A = \Im \left( {y^{\prime\prime}_{0} + p\left( { - y^{\prime\prime}_{0} - 3y^{2} - 2x^{2} y} \right)} \right) , $$

where, we defined $A = y^{\prime}(0)$.

Solving for $Y(s)$ and applying Laplace inverse transform $\Im^{ - 1}$

53 $$ y(x) = \Im^{ - 1} \left\{ {\frac{A}{{s^{2} }} + \frac{1}{{s^{2} }}\left( {\Im \left( {y^{\prime\prime}_{0} + p\left( { - y^{\prime\prime}_{0} - 3y^{2} - 2x^{2} y} \right)} \right)} \right)} \right\} $$

Next, we assume the solution for (46) has the form

54 $$ y(x) = \sum\limits_{n = 0}^{\infty } {p^{n} } \nu_{n} (x) , $$

and we choose

55 $$ y_{0} (x) = Ax , $$

as the first approximation for the solution of (46) that satisfies the condition $y(0) = 0.$

After substituting (54) and (55) into (53), we get

56 $$ \sum\limits_{n = 0}^{\infty } {p^{n} \nu_{n} } = \Im^{ - 1} \left\{ {\frac{A}{{s^{2} }} + \frac{p}{{s^{2} }}\Im \left( { - 3\left( {\nu_{0} + p\nu_{1} + p^{2} \nu_{2} + \cdots} \right)^{2} - 2x^{2} \left( {\nu_{0} + p\nu_{1} + p^{2} \nu_{2} + \cdots} \right)} \right)} \right\} , $$

Equating terms with identical powers of $p$, we obtain

57 $$ p^{0} :\nu_{0} (x) = \Im^{ - 1} \left\{ {\frac{A}{{s^{2} }}} \right\}, $$

58 $$ p^{1} :\nu_{1} (x) = \Im^{ - 1} \left\{ {\left( {\frac{1}{{s^{2} }}} \right)\Im \left( { - 3\nu_{0}^{2} - 2x^{2} \nu_{0} } \right)} \right\} ,$$

59 $$ p^{2} :\nu_{2} (x) = \Im^{ - 1} \left\{ {\left( {\frac{1}{{s^{2} }}} \right)\Im \left( { - 6\nu_{0} \nu_{1} - 2x^{2} \nu_{1} } \right)} \right\} , $$

… .

Solving the above equations for $$ \nu_{0} (x), $$$$ \nu_{1} (x), $$$$ \nu_{2} (x),\ldots $$ we obtain

60 $$ p^{0} :\nu_{0} (x) = Ax, $$

61 $$ p^{1} :\nu_{1} (x) = \frac{{ - A^{2} }}{4}x^{4} - \frac{A}{10}x^{5} , $$

62 $$ \begin{aligned} &p^{2} :\nu_{2} (x) = \frac{{A^{3} }}{28}x^{7} + \frac{{11A^{2} }}{560}x^{8} + \frac{A}{360}x^{9} ,\\  &\quad \ldots,\end{aligned} $$

and so on.

By substituting (60)–(62) into (54) and calculating the limit when $p \to 1$, results in a second order approximation

63 $$ y(x) = Ax - \frac{{A^{2} }}{4}x^{4} - \frac{A}{10}x^{5} + \frac{{A^{3} }}{28}x^{7} + \frac{{11A^{2} }}{560}x^{8} + \frac{A}{360}x^{9} . $$

In order to calculate the value of $A$, we require that (63) satisfies the boundary condition $y(1) = 1/2$, so that we obtain

64 $$ A = 0.651098543. $$

Thus, substituting (64) into (63), we obtain a handy LT-HPM solution.

65 $$ \begin{aligned} y(x) &= 0.651098543x - 0.1059823282x^{4} - 0.06510985430x^{5} \\ & \quad + 0.009857848493x^{7} + 0.008327182928x^{8} + 0.001808607064x^{9}. \end{aligned} $$

### Case 2

This case study employs CVLT-HPM method.

We will obtain the exact solution for the problem of finding a function that extremizes the value of the integral.

66 $$ \int_{{x_{1} }}^{{x_{2} }} {\left( {\frac{1}{2}x^{2} y^{\prime 2} + y^{2} + 8x^{2} y} \right)} dx. $$

After employing Euler equation (2), for $f = \frac{1}{2}x^{2} y^{\prime 2} + y^{2} + 8x^{2} y$ we obtain

67 $$ x^{2} y^{\prime\prime}(x) + 2xy^{\prime}(x) - 2y(x) - 8x^{2} = 0. $$

Considering the boundary conditions $y(0) = 0$,$y(1) = 1$, our purpose will be to get an approximate solution for the following non homogeneous variable coefficients linear equation.

68 $$ x^{2} y^{\prime\prime}(x) + 2xy^{\prime}(x) - 2y(x) - 8x^{2} = 0,\quad y(0) = 0,\;y(1) = 1. $$

Since it is not known in advance the value of $y^{\prime}(0)$ and (67) has a term containing $y^{\prime}(x)$ [see “Basic idea of Laplace transform homotopy perturbation method with variable coefficients (CVLT-HPM)” section (2)], then, it is better to write (67) in a simpler way by using the following substitution (Simmons 1983)

69 $$ xy(x) = u(x), $$

this transforms an equation in the standard form as (67), in its normal form given by

70 $$ u^{\prime\prime}(x) + q(x)u(x) - 8x = 0, $$

where

71 $$ q(x) = \frac{ - 2}{{x^{2} }}. $$

Besides (69) implies the following boundary conditions for *u:*

72 $$ u(0) = 0,\,\,\,\,u(1) = 1, $$

since $$ y(0) = 0 $$ and $$ y(1) = 1. $$

Therefore, we can express formally our problem in terms of $u$ as follows

73 $$\begin{aligned} & u^{\prime\prime}(x) - \frac{2}{{x^{2} }}u(x) - 8x = 0, \\  &u(0) = 0,\,\,\,u(1) = 1.\end{aligned} $$

In the same way, we note that

$$ u^{\prime } (x) = xy^{\prime } (x) + y(x), $$

implies the condition

74 $$ u^{\prime } (0) = 0. $$

With the purpose of applying CVLT-HPM, we will rewrite (70) as follows

75 $$ xu^{\prime\prime} (x) - \frac{2}{x}u(x) - 8x^{2} = 0. $$

In accordance with “Basic idea of Laplace transform homotopy perturbation method with variable coefficients (CVLT-HPM)” section, it is convenient to construct the following homotopy.

76 $$ (1 - p)(xu^{{{\prime \prime }}} - xD) + p\left[ {xu^{\prime \prime } - \frac{2u}{x} - 8x^{2} } \right] = 0, $$

where we have chosen as trial function, the constant function $w(x) = D$.

In order to apply CVLT-HPM, we simplify (76) as follows

77 $$ xu^{\prime \prime } = xD + p\left[ { - xD + \frac{2u}{x} + 8x^{2} } \right]. $$

After applying Laplace transform to (77) we get

$$ \Im \left\{ {xu^{\prime \prime } } \right\} = \Im \left\{ {xD + p\left[ { - xD + \frac{2u}{x} + 8x^{2} } \right]} \right\}. $$

As it is explained in (Spiegel 1998) it is possible to rewrite the above equation, as

78 $$ - \frac{d}{ds}\left( {s^{2} U(s)} \right) = \Im \left( {xD + p\left( { - xD + \frac{2u}{x} + 8x^{2} } \right)} \right), $$

where we have defined $U(s) = \Im (u(x))$ and employed the initial conditions $u(0) = 0$, and $u^{\prime}(0) = 0$ [see (73) and (74)].

After integrating (78), we obtain

79 $$ s^{2} U(s) = - \int {\Im \left\{ {xD + p\left[ { - xD + \frac{2u}{x} + 8x^{2} } \right]} \right\}ds} . $$

Applying $\Im^{ - 1}$ to this last integral expression, yields in

80 $$ u(x) = - \Im^{ - 1} \left\{ {\left( {\frac{1}{{s^{2} }}} \right)\int {\Im \left\{ {xD + p\left[ { - xD + \frac{2u}{x} + 8x^{2} } \right]} \right\}ds} } \right\}. $$

Next, we assume a series solution for $y(x)$, in the form

81 $$ u(x) = \sum\limits_{n = 0}^{\infty } {p^{n} } \nu_{n} , $$

Substituting (81) into (80), we get

82 $$ \sum\limits_{n = 0}^{\infty } {p^{n} } \nu_{n} = \Im^{ - 1} \left\{ {\left( {\frac{1}{{s^{2} }}} \right)\int {\Im \left\{ { - xD + p\left[ {xD - \frac{{2\sum\limits_{n = 0}^{\infty } {p^{n} } \nu_{n} }}{x} - 8x^{2} } \right]} \right\}ds} } \right\}. $$

On comparing the coefficients with identical powers of $$ p, $$ and perform the indicated operations, we have

83 $$ p^{0} :\nu_{0} = \Im^{ - 1} \left\{ {\left( {\frac{1}{{s^{2} }}} \right)\int {\Im \left\{ { - xD} \right\}ds} } \right\}, $$

84 $$ p^{1} :\nu_{1} = \Im^{ - 1} \left\{ {\left( {\frac{1}{{s^{2} }}} \right)\int {\Im \left\{ {xD - \frac{{2\nu_{0} }}{x} - 8x^{2} } \right\}ds} } \right\}, $$

85 $$ p^{2} :\nu_{2} = \Im^{ - 1} \left\{ {\left( {\frac{1}{{s^{2} }}} \right)\int {\Im \left\{ {\frac{{ - 2\nu_{1} }}{x}} \right\}ds} } \right\}, $$

86 $$ p^{3} :\nu_{3} = \Im^{ - 1} \left\{ {\left( {\frac{1}{{s^{2} }}} \right)\int {\Im \left\{ {\frac{{ - 2\nu_{2} }}{x}} \right\}ds} } \right\}, $$

87 $$ p^{4} :\nu_{4} = \Im^{ - 1} \left\{ {\left( {\frac{1}{{s^{2} }}} \right)\int {\Im \left\{ {\frac{{ - 2\nu_{3} }}{x}} \right\}ds} } \right\}, $$

… .

Solving the above operations for $$ \nu_{0} (x), $$$$ \nu_{1} (x), $$$$ \nu_{2} (x)\ldots $$ we obtain

88 $$ p^{0} :\nu_{0} (x) = \frac{D}{2}x^{2}, $$

89 $$ p^{1} :\nu_{1} (x) = \frac{{4x^{3} }}{3}, $$

90 $$ p^{2} :\nu_{2} (x) = \frac{{4x^{3} }}{(3)(3)}, $$

91 $$ p^{3} :\nu_{3} (x) = \frac{{4x^{3} }}{(3)(3)(3)}, $$

92 $$\begin{aligned} p^{4} :\nu_{4} (x) &= \frac{{4x^{3} }}{(3)(3)(3)(3)}, \\ &\quad \ldots ,\end{aligned} $$

93 $$\begin{aligned} p^{n} :\nu_{n} (x) &= \frac{{4x^{3} }}{(3)(3)(3)(3)\ldots (3)(3)} = \frac{{4x^{3} }}{{(3)^{n} }},\\  &\quad \ldots, \end{aligned}$$

and so on.

By substituting solutions (88)–(93) into (81) and calculating the limit value $p \to 1$, results in.

94 $$ u(x) = \frac{D}{2}x^{2} + \frac{4}{3}\left[ {1 + \frac{1}{3} + \left( {\frac{1}{3}} \right)^{2} + \left( {\frac{1}{3}} \right)^{3} + \cdots + \left( {\frac{1}{3}} \right)^{n} } \right]x^{3} . $$

Clearly, the second term of the right hand side of (94) is a geometric series of ratio $r = 1/3$. Since $\left| r \right| < 1$, it is well known that the mentioned series converges and the sum of the first n terms is given by $\frac{{1 - \left( {1/3} \right)^{n} }}{1 - 1/3}$ (Simmons 1983); therefore (94) becomes in

95 $$ u(x) = \frac{D}{2}x^{2} + 2\left[ {1 - \left( {1/3} \right)^{n} } \right]x^{3} . $$

Assuming that $n \to \infty$, we get

96 $$ u(x) = \frac{D}{2}x^{2} + 2x^{3} . $$

From the boundary condition $y(1) = 1$ we get $D = - 2$, therefore

97 $$ u(x) = - x^{2} + 2x^{3} . $$

Finally, from (69) we obtain the solution for the boundary value problem (68)

98 $$ y(x) = - x + 2x^{2} . $$

Thus, for this case study CVLT-HPM provides an exact solution for (68).

### Case 3

Next, we will analyze the case of

99 $$ \int_{{x_{1} }}^{{x_{2} }} {\frac{{\sqrt {1 + y^{\prime 2} } }}{{y^{1/n} }}} dx. $$

After employing Euler equation (8), we get

100 $$ y^{2/n} (x)(1 + y^{\prime 2} (x)) = c $$

where $c$ is a constant.

Later, we will require to express (100) in such a way that it does not involves $c.$ Therefore we differentiate (100) respect to $x$.

101 $$ \frac{{d\left( {y^{2/n} (x)(1 + y^{\prime 2} (x))} \right)}}{dx} = 0. $$

We rewrite (100) as follows

102 $$ y^{2} y^{\prime 2} = cy^{{2\left( {1 - \frac{1}{n}} \right)}} - y^{2} , $$

or

103 $$ \left( {\frac{{dy^{2} }}{dx}} \right)^{2} = 4\left[ {cy^{{2\left( {1 - \frac{1}{n}} \right)}} - y^{2} } \right], $$

so that

104 $$ y^{2\prime } = 2{\sqrt{c}} y^{{\left( {\frac{n - 1}{n}} \right)}} \left[ {1 - \frac{{y^{2/n} }}{c}} \right]^{1/2} , $$

where we employed the notation

105 $$ y^{2 \prime} = \left( {\frac{{dy^{2} }}{dx}} \right). $$

Next, we propose the following substitution

106 $$ y = u^{n/n - 1} ,\quad n \ne 1. $$

Thus, (104) adopts the form

107 $$ \frac{{du^{{\frac{2n}{n - 1}}} }}{dx} = 2{\sqrt{c}} u\left[ {1 - \frac{{u^{2/n - 1} }}{c}} \right]^{1/2} .\,\,n \ne 1. $$

We will consider as a case study $n = 3$. It corresponds to the equation (see 100) (Filobello-Nino et al. 2013d).

108 $$ y^{2/3} (x)(1 + y^{\prime 2} (x)) = c. $$

We note, it is possible to rewrite (108) as

109 $$ x = \int {\frac{{y^{1/3} dy}}{{\sqrt {c - y^{2/3} } }}} + c. $$

Although (109) can be solved performing the algebraic substitution $y = v^{3}$, the dependent variable $y(x)$ remains implicit.

In fact, we propose the following boundary value problem.

110 $$ \begin{aligned} & y^{2/3} (x)(1 + y^{\prime 2} (x)) = c, \\ &y(0) = 0,\quad y(20) = 1. \end{aligned} $$

In order to get an explicit analytical approximate solution for (110), we will consider (106) and (107) for $n = 3$.

111 $$ y(x) = u^{3/2} (x), $$

112 $$ \frac{{du^{3} }}{dx} = 2{\sqrt{c}} u\left[ {1 - \frac{u}{c}} \right]^{1/2} . $$

Next, we use Newton’s binomial to transform (112) into the following approximate equation

113 $$ \frac{{du^{3} }}{dx} - 2{\sqrt{c}} u + \frac{{u^{2} }}{\sqrt{c}} + \frac{{u^{3} }}{{4c^{3/2} }} = 0. $$

Such as it will be explained in discussion section, although LT-HPM is an appropriate formulation for this case study, it is required a reformulation because the term $2{\sqrt{c}} u$, gives rise to an approximate solution inconsistent with the proposed boundary condition $u(0) = 0$(from condition (111) we see that $y(0) = 0 \to u(0) = 0$). In order to delay the effect of the above mention term in the iterative process, we begin proposing the following homotopy, which is an original contribution of this work (see also, “Discussion” section).

114 $$ (1 - p^{n} )({{du^{3} } /{dx}} - w) + p^{n} \left[ {{{du^{3} } / {dx}} + {{u^{3} }/{4c^{3/2} }} + {{u^{2} } {\sqrt c - 2\sqrt c u}}} \right] = 0, $$

where we have introduced a trial function $w$ which may depend, for our purposes of

115 $$ w = w(x,u,p), $$

and $n$ is an integer number $n > 1$.

We rewrite the above equation as follows.

116 $$ \frac{{du^{3} }}{dx} = w + p^{n} \left[ { - w - {{u^{3} } / {4c^{3/2} }} - {{u^{2} } / {{\sqrt {c}} + 2{\sqrt {c}} u}}} \right]. $$

Next, using NDLT-HPM we propose

117 $$ w = 3Ax^{2} - \frac{{u^{3} p}}{{4c^{3/2} }} - \frac{{u^{2} p}}{{c^{1/2} }}, $$

so that (116) becomes

118 $$ \frac{{du^{3} }}{dx} = 3Ax^{2} - {{u^{3} p} / {4c^{3/2} }} - {{u^{2} p} / {{\sqrt {c}} }} + p^{n} \left[ { - 3Ax^{2} + {{u^{3} (p - 1)} /{4c^{3/2} }} + {{u^{2} (p - 1)} /{{\sqrt {c}}+ 2{\sqrt {c}}u}}} \right]. $$

It is worthwhile to mention, after evaluating the limit $p \to 1$, we obtain the equation to solve (116) from (118) as it should be if (118) has to be a valid homotopy.

After considering $n$ values as large as we wish ($n \to \infty$), it is clear that for practical purposes, (118) is equivalent to

119 $$ \frac{{du^{3} }}{dx} = 3Ax^{2} - {{u^{3} p} / {4c^{3/2} }} - {{u^{2} p} /{{\sqrt {c}} }}. $$

Thus, (118) let us to delay sufficiently the role of the term $2{\sqrt {c}} u$ in the iterative process and replacing it for $3Ax^{2}$; it eliminates the mentioned problem because the iterative process resulting will be consistent with $u(0) = 0$. We will show the calculation of parameter $A$ will allow recover part of lost information for the omission of term $2{\sqrt {c}} u$, and the substitution of (113) instead of (112).

Following the usual Laplace transform algorithm, applying $\Im$ to (119) we obtain

120 $$ \Im \left\{ {\frac{{du^{3} }}{dx}} \right\} = \Im \left\{ {3Ax^{2} - {{u^{3} p} / {4c^{3/2} }} - {{u^{2} p} / {{\sqrt {c}} }}} \right\}. $$

As it is explained in (Spiegel 1998) we rewrite (120) as follows

121 $$ s\Im \left\{ {u^{3} } \right\} = \Im \left\{ {3Ax^{2} - {{u^{3} p} /{4c^{3/2} }} - {{u^{2} p} / {{\sqrt {c}} }}} \right\} , $$

where we have employed the initial condition $u(0) = 0$.

Applying Laplace inverse transform $\Im^{ - 1}$ to this last expression, we obtain

122 $$ u^{3} = \Im^{ - 1} \left\{ {\left( {1/s} \right)\Im \left\{ {3Ax^{2} - {{u^{3} p} / {4c^{3/2} }} - {{u^{2} p} /{{\sqrt {c}} }}} \right\}} \right\} $$

Next, we assume a series solution for $u(x)$, in the form

123 $$ u(x) = \sum\limits_{n = 0}^{\infty } {p^{n} } \nu_{n} . $$

Substituting (123) into (122), we get

124 $$ \left( {\sum\limits_{n = 0}^{\infty } {p^{n} \nu_{n} } } \right)^{3} = \Im^{ - 1} \left\{ {\left( {1/s} \right)\Im \left\{ {3Ax^{2} - {{\left( {\sum\limits_{n = 0}^{\infty } {p^{n} \nu_{n} } } \right)^{3} p} / {4c^{3/2} }} - p{{\left( {\sum\limits_{n = 0}^{\infty } {p^{n} \nu_{n} } } \right)^{2} } /{{\sqrt {c}} }}} \right\}} \right\}. $$

After comparing the coefficients of like powers of $$ p, $$ and perform the indicated operations, we have

125 $$ p^{0} :\nu_{0}^{3} = \Im^{ - 1} \left\{ {\left( {\frac{1}{s}} \right)\Im \left\{ {3Ax^{2} } \right\}} \right\}, $$

126 $$ p^{1} :\nu_{1} \nu_{0}^{2} + 2\nu_{1} \nu_{0}^{2} = \Im^{ - 1} \left\{ {\left( {\frac{1}{s}} \right)\Im \left\{ {\left[ { - \nu_{0}^{3} /4c^{3/2} - \nu_{0}^{2} /{\sqrt {c}} } \right]} \right\}} \right\}, $$

127 $$ p^{2} :3\nu_{2} \nu_{0}^{2} + 2\nu_{0} \nu_{1}^{2} = \Im^{ - 1} \left\{ {\left( {\frac{1}{s}} \right)\Im \left\{ {\left[ { - 3\nu_{1} \nu_{0}^{2} /4c^{3/2} - 2\nu_{1} \nu_{0} /{\sqrt {c}} } \right]} \right\}} \right\}, $$

128 $$ p^{3} :\nu_{0}^{2} \nu_{3} + \nu_{1}^{3} + 6\nu_{0} \nu_{1} \nu_{2} = \Im^{ - 1} \left\{ {\left( {\frac{1}{s}} \right)\Im \left\{ {\left[ { - (3\nu_{2} \nu_{0}^{2} + 3\nu_{1}^{2} \nu_{0} )/4c^{3/2} - (2\nu_{2} \nu_{0} + \nu_{1}^{2} )/{\sqrt {c}} } \right]} \right\}} \right\}. $$

Solving the above operations for $$ \nu_{0} (x), $$$$ \nu_{1} (x), $$ and $$ \nu_{2} (x), $$ we obtain

129 $$ p^{0} :\nu_{0} (x) = A^{1/3} x, $$

130 $$ p^{1} :\nu_{1} (x) = \frac{{ - A^{1/3} x^{2} }}{{48c^{3/2} }} - \frac{x}{9{\sqrt {c}} }, $$

131 $$ p^{2} :\nu_{2} (x) = \frac{{13A^{1/3} }}{{17280c^{3} }}x^{3} + \frac{{19x^{2} }}{{2592c^{2} }} + \frac{4}{{243A^{1/3} c}}x, $$

132 $$ \begin{aligned}p^{3} :\nu_{3} (x) &= - \frac{{10395Ax^{4} + 202608x^{3} cA^{2/3} + 345600x^{2} c^{2} A^{1/3} + 102400xc^{3} }}{{74649600c^{9/2} A^{2/3} }},\\ &\ldots,\end{aligned} $$

and so on.

By substituting (129)–(132) into (123), calculating the limit when $p \to 1$, we obtain the following third order approximation.

133 $$ \begin{aligned} u(x) &= \frac{{13A^{1/3} }}{{17280c^{3} }}x^{3} + \left( {\frac{{ - A^{3} }}{{48c^{3/2} }} + \frac{19}{{2592c^{2} }}} \right)x^{2} + \left( {A^{1/3} - \frac{1}{9{\sqrt {c}} } + \frac{4}{{243A^{1/3} c}}} \right)x + \frac{1}{{A^{2/3} x^{2} }}\left( {\frac{{A^{1/3} x^{2} }}{{48c^{3/2} }} - \frac{x}{9{\sqrt {c}} }} \right)^{3} - \frac{37}{{14400c^{7/2} }}x^{3}\\ &\quad -\frac{7}{{1296c^{5/2} A^{1/3} }}x^{2} - \frac{{41A^{1/3} }}{{276480c^{9/2} }}x^{4}. \end{aligned} $$

From (111) and (133) we obtain

134 $$ y(x) = \left( {\frac{{13A^{1/3} }}{{17280c^{3} }}x^{3} + \left( {\frac{{ - A^{3} }}{{48c^{3/2} }} + \frac{19}{{2592c^{2} }}} \right)x^{2} + \left( {A^{1/3} - \frac{1}{9{\sqrt {c}} } + \frac{4}{{243A^{1/3} c}}} \right)x + \frac{1}{{A^{2/3} x^{2} }}\left( {\frac{{A^{1/3} x^{2} }}{{48c^{3/2} }} - \frac{x}{9{\sqrt {c}} }} \right)^{3} - \frac{37}{{14400c^{7/2} }}x^{3} - \frac{7}{{1296c^{5/2} A^{1/3} }}x^{2} - \frac{{41A^{1/3} }}{{276480c^{9/2} }}x^{4} } \right)^{3/2} . $$

In order to calculate the values of $$ A $$ and $$ c, $$ we will require that (134) satisfies the proposed boundary condition $$ y(20) = 1 $$. Also, proceeding as it is indicated in “Basic idea of Laplace transform homotopy perturbation method with variable coefficients (CVLT-HPM)” section, we substitute (134) into (101) for $$ n=3 $$ and evaluating the expression so obtained for some value $$ x $$. Choosing $$ x = 19.8 $$, which lies into the interval of interest, $$ 0 \le x \le 20 $$(see “Discussion” section below), we obtain an system of algebraic equations for $$ A $$ and $$ c $$, whose solution is given by:

135 $$ A = 0.001806042527,\,\,c = 3.428590549. $$

After substituting (135) into (134) we obtain the following approximation

136 $$ y(x) = \left( \begin{aligned}&- 0.00003215665052x^{3} - 0.001414069432x^{2} + 0.1011974436x \hfill \\ &\quad + \frac{{(67.42920175)\left( {0.0003996329765x^{2} - 0.06000669099x} \right)^{3} }}{{x^{2} }} - 7.057905179 \times 10^{ - 8} x^{4} \end{aligned}\right)^{3/2} $$

### Case 4

This case is studied, by using a combination of LT-HPM and NDLT-HPM.

Finally we will consider the problem of extremizing an integral of the form

137 $$ \int_{{x_{1} }}^{{x_{2} }} {\frac{{\sqrt {1 + y^{\prime 2} } }}{g(x)}} dx, $$

where, $g(x)$ is an arbitrary function, except having Taylor series.

Defining $f(x) = 1/g(x)$, it is clear that (f1) is formally equivalent to the case

138 $$ \int_{{x_{1} }}^{{x_{2} }} {f(x)\sqrt {1 + y^{\prime 2} } } dx. $$

After employing Euler equation (7), for $f = \frac{{\sqrt {1 + y^{\prime 2} } }}{g(x)},$ we obtain the following differential equation

139 $$ y^{\prime}(x) = \frac{cg(x)}{{\sqrt {1 - c^{2} g^{2} (x)} }}, $$

where $c$ is a constant.

Separating variables, we rewrite (139) in terms of

140 $$ y = \int {\frac{cg(x)dx}{{\sqrt {c - c^{2} g^{2} (x)} }}} + C. $$

Unfortunately the above integral hardly can be solved in terms of elementary functions for many $g(x)$ functions.

In principle it is possible to provide an scheme in order to obtain analytical approximate solutions for problems like (139), by using LT-HPM.

After multiplying by $y(x)$, it is possible to rewrite (139) as

141 $$ \frac{{dy^{2} }}{dx} = \frac{2cgy}{{\sqrt {1 - c^{2} g^{2} } }} = 2cgy\left( {1 - c^{2} g^{2} } \right)^{ - 1/2}. $$

We employ Newton’s binomial to transform (141) into the following approximate equation

142 $$ \frac{{dy^{2} }}{dx} = 2cgy\left( {1 + \frac{1}{2}c^{2} g^{2} + \frac{3}{8}c^{4} g^{4} } \right). $$

Following “Basic idea of non-linearities distribution Laplace transform-homotopy perturbation method (NDLT-HPM)” and “Basic idea of laplace transform homotopy perturbation method with variable coefficients (CVLT-HPM)” sections, it is convenient to construct a homotopy as follows

143 $$ (1 - p)({{dy^{2} } / {dx}} - 2Ax) + p\left[ {{{dy^{2} } / {dx}} - 2cg(x,p)y - c^{3} g^{3} (x,p)y - 3c^{5} g^{5} (x,p)y/4} \right] = 0, $$

where in one hand we have chosen as a polynomial trial function $$ w(x) = 2Ax, $$ (*A* is a parameter to be determined) and on the other hand we emphasized the possibility of embedded the homotopy parameter $$ p $$ into the function $$ g(x) $$ (see “Discussion” section).

Next we rewrite the above equation as

144 $$ \frac{{dy^{2} }}{dx} = 2Ax + p\left[ { - 2Ax + 2cyg(x,p) + c^{3} g^{3} (x,p)y + 3c^{5} g^{5} (x,p)y/4} \right]. $$

Following Laplace transform algorithm, we apply $\Im$

145 $$ \Im \left\{ {\frac{{dy^{2} }}{dx}} \right\} = \Im \left\{ {2Ax + p\left[ { - 2Ax + 2cyg(x,p) + c^{3} g^{3} (x,p)y + 3c^{5} g^{5} (x,p)y/4} \right]} \right\}. $$

Such as it was explained in (Spiegel 1998) we rewrite (145) as follows

146 $$ s\Im \left\{ {y^{2} } \right\} - y(0) = \Im \left\{ {2Ax + p\left[ { - 2Ax + 2cyg(x,p) + c^{3} g^{3} (x,p)y + 3c^{5} g^{5} (x,p)y/4} \right]} \right\}. $$

Applying Laplace inverse transform $\Im^{-1}$ to the above expression we get

147 $$ y^{2} = \Im^{ - 1} \left\{ {\frac{y(0)}{s} + \frac{\Im }{s}\left\{ {2Ax + p\left[ { - 2Ax + 2cyg(x,p) + c^{3} g^{3} (x,p)y + 3c^{5} g^{5} (x,p)y/4} \right]} \right\}} \right\}. $$

Next, in order to show how to work the methodology indicated by (147), we propose as cases: $$ g(x) = \exp (x); $$ with two different boundary conditions $$ y(0) = 0 , $$$$ y(1) = 1/2 $$ and $$ y(0) = 0 $$,$$ y(1) = 1 $$ in order to show the flexibility of the proposed methodology. So that for instance, we can express formally (139) for our first case as follows

148 $$ y^{\prime}(x) = \frac{c\exp (x)}{{\sqrt {1 - c^{2} \exp (2x)} }}\quad y(0) = 0,\,\,\,y(1) = 1/2, $$

in the same manner after, we will consider the case $y(0) = 0$,$y(1) = 1$.

Later, we will require expressing (148) in such a way that it does not involves $c.$ For that purpose we obtain the following equivalent expression for (148)

$$ \frac{{y^{\prime}(x)}}{{\exp (x)\sqrt {1 + y^{\prime}(x)^{2} } }} = c, $$

differentiating respect to $x$, in order to eliminate $c$ we get

149 $$ \frac{{d\left( {\frac{{y^{\prime}(x)}}{{\exp (x)\sqrt {1 + y^{\prime}(x)^{2} } }}} \right)}}{dx} = 0. $$

Thus, the proposed analytical approximate solution (147), adopts the form

150 $$ y^{2} = \Im^{ - 1} \left\{ {\frac{\Im }{s}\left\{ {2Ax + p\left[ { - 2Ax + 2cy\exp (px) + c^{3} y\exp (3px) + 3c^{5} y\exp (5px)/4} \right]} \right\}} \right\}. $$

Next, we assume a series solution for $y(x)$, in the form

151 $$ y(x) = \sum\limits_{n = 0}^{\infty } {p^{n} } \nu_{n}. $$

In order to keep control of the different $p$ order approximations from (150), we introduce the following Taylor series expansions.

$$ \exp (px) = 1 + px + \frac{{p^{2} x^{2} }}{2} + \cdots $$

$$ \exp (3px) = 1 + 3px + \frac{{9p^{2} x^{2} }}{2} + \cdots , $$

152 $$ \exp (5px) = 1 + 5px + \frac{{25p^{2} x^{2} }}{2} + \cdots . $$

Substituting (151) and (152) into (150), we get

153 $$ \left( {\sum\limits_{n = 0}^{\infty } {p^{n} \nu_{n} } } \right)^{2} = \Im^{ - 1} \left\{ {\frac{\Im }{s}\left\{ {2Ax + p\left[ { - 2Ax + 2c\left( {\sum\limits_{n = 0}^{\infty } {p^{n} \nu_{n} } } \right)\left( {1 + px + \frac{{p^{2} x^{2} }}{2} + \cdots } \right) + c^{3} \left( {\sum\limits_{n = 0}^{\infty } {p^{n} \nu_{n} } } \right)\left( {1 + 3px + \frac{{9p^{2} x^{2} }}{2} + \cdots } \right) + 3c^{5} \left( {\sum\limits_{n = 0}^{\infty } {p^{n} \nu_{n} } } \right)\left( {1 + 5px + \frac{{25p^{2} x^{2} }}{2} + \cdots } \right)/4} \right]} \right\}} \right\}. $$

On comparing the coefficients of like powers of $$ p, $$ we have

154 $$ p^{0} :\nu_{0}^{2} = \Im^{ - 1} \left\{ {\frac{1}{s}\Im (2Ax)} \right\}, $$

155 $$ p^{1} : 2\nu_{0} \nu_{1} = \Im^{ - 1} \left\{ {\left( {\frac{1}{s}} \right)\Im \left\{ {\left[ { - 2Ax + 2c\nu_{0} + c^{3} \nu_{0} + 3c^{5} \nu_{0} /4} \right]} \right\}} \right\}, $$

156 $$ p^{2} : \nu_{1}^{2} + 2\nu_{0} \nu_{2} = \Im^{ - 1} \left\{ {\left( {\frac{1}{s}} \right)\Im \left\{ {\nu_{1} \left( {2c + c^{3} + 3c^{5} /4} \right) + \nu_{0} x\left( {2c + 3c^{3} + 15c^{5} /4} \right)} \right\}} \right\} , $$

157 $$ \begin{aligned} p^{3}: 2\nu_{0} \nu_{3} + 2\nu_{1} \nu_{2} &= \Im^{ - 1} \left\{ {\left( {\frac{1}{s}} \right)\Im \left\{ {\nu_{2} \left( {2c + c^{3} + \frac{3}{4}c^{5} } \right) + \nu_{1} x\left( {2c + 3c^{3} + \frac{{15c^{5} }}{4}} \right) + \nu_{0} x^{2} \left( {c + \frac{{9c^{3} }}{2} + \frac{{75c^{5} }}{8}} \right)} \right\}} \right\}, \\ &\quad  \ldots.\end{aligned} $$

After solving the above operations for $$ \nu_{0} (x), $$$$ \nu_{1} (x), $$ and $$ \nu_{2} (x) $$ we obtain

158 $$ p^{0} :\nu_{0} (x) = {\sqrt {A}} x, $$

159 $$ p^{1} :\nu_{1} (x) = \left[ { - \frac{{\sqrt {A}} }{2} + \frac{c}{2} + \frac{{c^{3} }}{4} + \frac{{3c^{5} }}{16}} \right]x, $$

160 $$ p^{2} :\nu_{2} (x) = \left[ { - \frac{{\sqrt {A}} }{2} + \frac{c}{2} + \frac{{c^{3} }}{4} + \frac{{3c^{5} }}{16}} \right]\left[ {\frac{{\sqrt {A}} }{2} + \frac{c}{2} + \frac{{c^{3} }}{4} + \frac{{3c^{5} }}{16}} \right]x + \frac{1}{6}\left( {2c + 3c^{3} + \frac{15}{4}c^{5} } \right)x^{2} , $$

161 $$ \begin{aligned} p^{3} :\nu_{3} (x) &= \frac{1}{8}\left( {c + \frac{{9c^{3} }}{2} + \frac{{75c^{5} }}{8}} \right)x^{3} + \frac{1}{6{\sqrt {A}} }\left[ {2c + 3c^{3} + \frac{{15c^{5} }}{4}} \right]\left[ {\frac{c}{3} + \frac{{c^{3} }}{6} + \frac{{c^{5} }}{8}} \right]x^{2} + \left( {\frac{ - {\sqrt {A}} }{2} + \frac{c}{2} + \frac{{c^{3} }}{4} + \frac{{3c^{5} }}{16}} \right)\left( {\frac{{\sqrt {A}} }{2} + \frac{c}{2} + \frac{{c^{3} }}{4} + \frac{{3c^{5} }}{16}} \right)\frac{x}{2}, \\ & \quad \ldots, \end{aligned}$$

and so on.

By substituting (158)–(161) into (151) and calculating the limit when $p \to 1$, results in a handy third order approximation

162 $$ y(x) = \frac{1}{8}\left( {c + \frac{{9c^{3} }}{2} + \frac{{75c^{5} }}{8}} \right)x^{3} + \frac{1}{6}\left[ {2c + 3c^{3} + \frac{{15c^{5} }}{4}} \right]\left[ {1 + \frac{1}{{\sqrt {A}} }\left( {\frac{c}{3} + \frac{{c^{3} }}{6} + \frac{{c^{5} }}{8}} \right)} \right]x^{2} + \left[ {{\sqrt {A}} + \left( {\frac{ - {\sqrt {A}} }{2} + \frac{c}{2} + \frac{{c^{3} }}{4} + \frac{{3c^{5} }}{16}} \right)\left( {1 + \frac{3}{2}\left( {\frac{{\sqrt {A}} }{2} + \frac{c}{2} + \frac{{c^{3} }}{4} + \frac{{3c^{5} }}{16}} \right)} \right)} \right]x. $$

In order to calculate the values of $$ A $$ and $$ c, $$ we will require that (162) satisfies the boundary condition $$ y(1) = 1/2. $$ Also, following “Basic idea of Laplace transform homotopy perturbation method with variable coefficients (CVLT-HPM)” section, we substitute (162) into (149) and evaluate the expression so obtained for some value, let us say $$ x = 1/5 $$, which lies into the interval of interest,$$ 0 \le x \le 1 $$(see “Discussion” below). After performing the above substitution, we obtain a system of algebraic equations for $$ A $$ and $$ c $$, whose solution provides

163 $$ A = 1.889510047,\quad c = 0.3720801807 $$

Thus, substituting (163) into (162), we obtain a handy approximate solution for (158)

164 $$ y(x) = 0.2474371491x + 0.1687199868x^{2} + 0.08384286388x^{3} . $$

Proceeding the same manner for the conditions, $$ y(0) = 0 $$, $$ y(1) = 0.7, $$ we obtain the values $$ A = 0.002029581087 $$, $$ c = 0.3131923646 $$. Finally, substituting them into (162), we get

165 $$ y(x) = 0.2267590836x + 0.4132800611x^{2} + 0.05996085479x^{3} . $$

## Discussion

In order to use a pure numerical solution as reference, we utilized the scheme based on trapezoid combined with Richardson extrapolation (Ascher et al. 1995; Ascher and Petzold 1998) from the built-in numerical routines provided by Maple 15. Moreover, the command was setup with an absolute error (AE) tolerance of $1 \times 10^{-12}$.

This work proposed LT-HPM and some its modifications, in order to find analytical approximate expressions for stationary functions solutions of the Euler equations, which turned out from four cases studied. Such as it was already mention, research in this area of knowledge is relevant because calculus of variations is a branch of the analysis with applications for both, pure and applied mathematics and expressing the basic principles of physics with simplicity and elegance among others.

We noted Euler equation (2) which describes these problems is of nonlinear nature and therefore the task of finding analytical solutions for this equation is in general a very difficult task. As a matter of fact, although some cases admit exact solutions, many variational problems admit just numerical solutions (Razzaghi and Elnagar 1994; Razzaghi and Yousefi 2001). Nevertheless this article showed one case study for which was not possible to find a numerical solution. Unlike (Lakestani and Kazemian 2013) where just one particular case of variational problem was solved, the goal of this paper was expose how LT-HPM and some of its modifications are employed in order to analyze a wide variety of cases study. Indeed, cases study 3 and 4 were presented in the most general way possible and to achieve it, we employed some modified versions of LT-HPM.

**Fig. 1:**
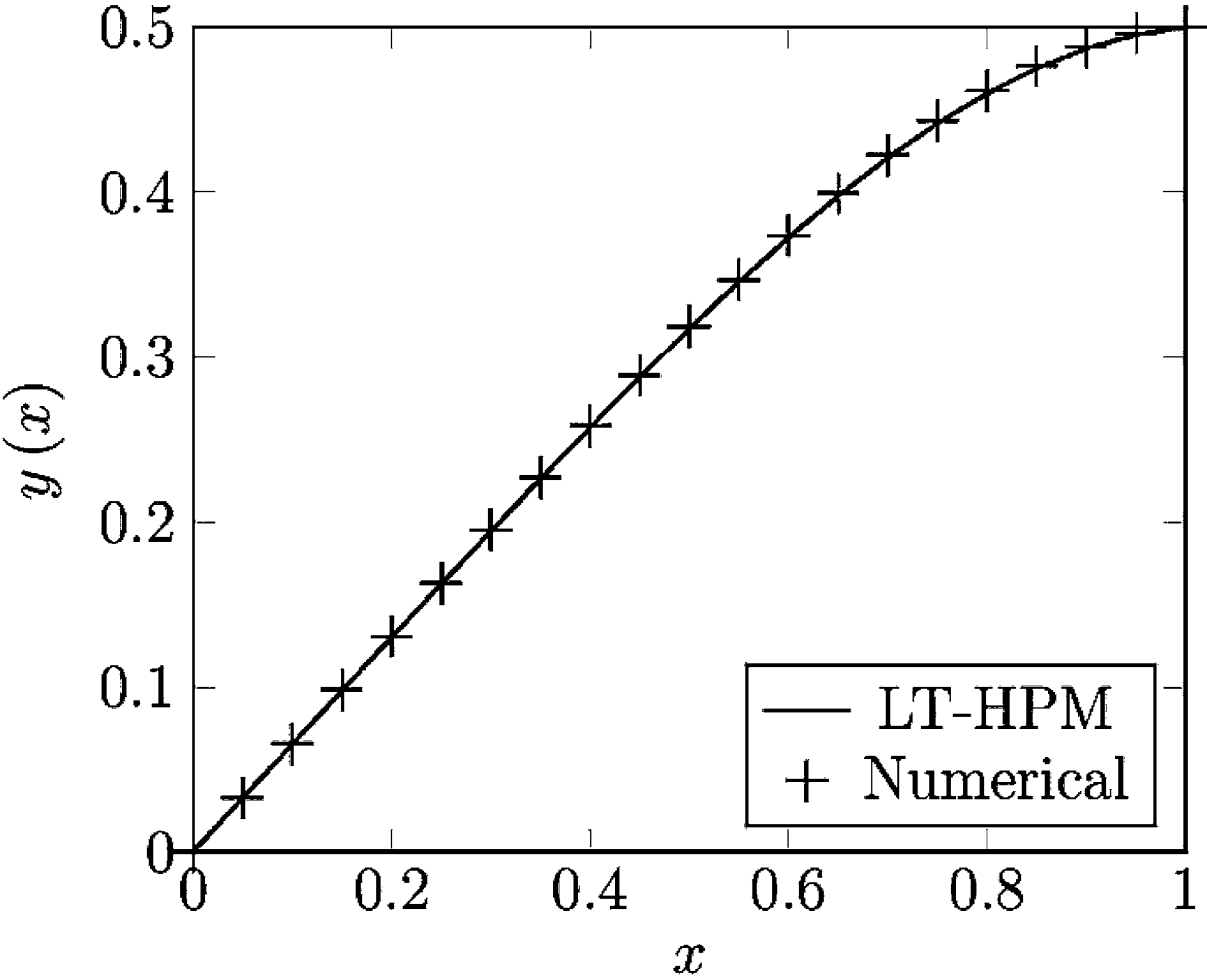
Comparison between numerical solution of (46) (*cross*) and LT-HPM approximation (65) (*line*)

Thus, case study 1 proposed the basic version of LT-HPM (“Basic idea of the LT-HPM method” section) in order to find an analytical approximate solution for (46); essentially we used the procedure followed in (Filobello-Nino et al. 2013c). The proposed method expresses the problem of solving a nonlinear differential equation in terms of solving an algebraic equation for some unknown initial condition [see (64)]. This formulation has shown dependable performance for the case of differential equations without the presence of singular points and this case satisfies these requirements (Filobello-Nino et al. 2013c). Figure 1 shows the comparison between numerical solution of (46) and LT-HPM second order approximation (65). From this figure is clear the proposed solution provides a good approximation. In more precise terms, to verify the precision of (63) and (65) we calculate the square residual error (SRE) of (65), which is explained in “Appendix 2”. SRE is in general terms a positive number representative of the total error by using an approximate solution for a given problem (Marinca and Herisanu 2011). The resulting value for our case study was 0.001918936920, which confirms the accuracy of the proposed solution. If more accuracy has to be required, one must consider higher order approximations.

Our second variational problem (66) gave rise to the second order non homogeneous linear differential equation with variable coefficients (68). Since the aforementioned equation is something long, and following the indications of “Basic idea of Laplace transform homotopy perturbation method with variable coefficients (CVLT-HPM)” section, we ease the application of the proposed methodology, transforming (67) in its normal form (70), by using the substitution (69) (Simmons 1983). The equivalent equation (73) is also of variable coefficients; therefore we employed the modified version of LT-HPM explained in “Basic idea of Laplace transform homotopy perturbation method with variable coefficients (CVLT-HPM)” section (Filobello-Nino et al. 2015b). CVLT-HPM expresses the problem to be solved in terms of a differential equation for Laplace transform $$ u(s), $$ although it easily solvable [see (78)–(80)].

Once $u(s)$ was expressed in terms of $u(x)$, we assumed a series solution in the form (81). CVLT-HPM method, calculated the different approximate orders, expressing the n-th iterative process in terms of integrals of the lower order approximations, previously calculated [see (83)–(87)].

Unlike basic LT-HPM algorithm (“Basic idea of the LT-HPM method” section), CVLT-HPM employed its homotopy freedom in order to substitute $L(u_{0} )$ as it was originally defined by HPM method (“Standard HPM” section), for the polynomial trial function $w(x) = D$ (constant), whose value was finally determined from the boundary condition $y(1) = 1$. It is remarkable to note we obtained an exact solution for (98), through the convergent geometric series (94), which shows that in those cases where a problem has an exact solution, CVLT-HPM method is able to provide it.

Case study 3 was presented in the most general possible manner (99). This case is relevant because, such as it was explained in “Background” section, some values of $n$ corresponds to known cases. Thus,$n = 1/2$ defines the problem of brachistochrone (Connor and Robertson 1997, 1998; Filobello-Nino et al. 2013d) and $n = - 1$ the problem of finding the curve that generates the surface of revolution of smallest area (Simmons 1983).

The corresponding Euler equation (102), presents an additional challenge; the presence of a highly nonlinear term that corresponds to the multiplication of $$ y^{2} $$ by $$ y^{\prime 2} $$ which difficult the application of LT-HPM algorithm. To overcome these difficulties, we proposed the substitution (106) in order to obtain (107). Despite of this equation involved the derivative of a power of dependent variable $$ u^{2n/n - 1} , $$ the methodology explained following Eqs. (114)–(119), turned out adequate to obtain an accurate analytical approximate solution for the case study $$ n = 3 $$ [see (110), (111), and (112)]. A relevant point of the proposed method is, although (108) is easily expressed in terms of separated variables, and the resulting integral (109) can be solved performing the algebraic substitution $$ y = v^{3} $$, the dependent variable $$ y(x) $$ remains implicit, and therefore Newton–Raphson method would be required to obtain values of $$ y(x) $$, given some values of $$ x $$. In the sequel, this case study not even admits a numerical solution.

On the other hand, such as we mentioned, the formulation of LT-HPM would turn out to be inappropriate for this case, because the term $$ 2{\sqrt {c}} u $$, would give rise to an approximate solution inconsistent with the proposed boundary condition $$ u(0) = 0. $$ As a matter of fact, if trial function was chosen as $$ z(x) = 0 $$ then $$ \nu_{0} (x) = 0 $$, and the rest of the iterations would vanish. If trial function is $$ z(x) = A $$(constant), then the subsequent iterative process would give rise to fractional powers of $$ x $$(in fact $$ \nu_{1} (x) = \sqrt[3]{A}x^{1/3} $$). The problem of solutions containing these powers is that, its derivatives becomes infinite at x = 0, unlike the initial conditions of the problem. It is not difficult to verify that a similar problem would occur if the trial function were chosen as one of the following: $$ z(x) = Ax, $$$$ z(x) = Ax^{3} , $$$$ z(x) = Ax^{4}, \ldots $$ and so on. Only the case $$ z(x) = Ax^{2} , $$ provides a whole powers polynomial function consistent with $$ u(0) = 0. $$ With the purpose of delay the effect of the problematic term $$ 2{\sqrt {c}} u $$, and introduce in the iterative process the presence of term $$ Ax^{2} , $$ we proposed employ the homotopy equation (119). In order to motivate it, we proposed the homotopy (114), which allows introduce a trial function, even depending of the homotopy parameter $$ p $$, $$ w = w(x,u,p) $$ in order to reach our goals. Nevertheless, we note (114) is a valid homotopy because (113) is recovered in the limit $$ p \to 1 $$, as expected. In particular by choosing $$ w $$ in accordance with (117), let us employed for practical purposes, to Eq. (119) instead of (113) in the limit $$ n \to \infty . $$ The above is due to the homotopy freedom of (114), which allowed delay sufficiently the role of the term $$ 2{\sqrt {c}} u, $$ and essentially substituted it for $$ 3Ax^{2} $$; thus the iterative process resulted to be consistent with $$ u(0) = 0 $$. The proposed homotopy (114), which is employed in order to delay and substitute a problematic term of a differential equation, including the introduction of a trial function depending of parameter $$ p $$ [see (115) and (117)] is an original contribution of this work. A relevant fact of the proposed method is the determination of parameter $$ A $$ allowed recovers part of lost information for the omission of term $$ 2{\sqrt {c}} u $$, and the substitution of (113) instead of (112). Then LT-HPM provided a third order approximation for $$ u $$ (133), and through Eq. (111) an approximate solution (134) for $$ y(x) $$. In order to calculate the values of $$ A $$ and $$ c $$ we required one hand, (134) satisfied the proposed boundary condition $$ y(20) = 1 $$, and on the other hand we deduced another equation by substituting (134) into (101) for $$ n = 3 $$ and then, evaluating the expression so obtained for some value which lied into the interval of interest $$ 0 \le x \le 20 $$. It is important to notice that unlike the others cases study, this example proposed a nonlinear differential equation defined in an interval relatively large in order to show the flexibility of the proposed method. Further research is required to propose a systematic procedure to obtain suitable cancelling points. For instance, the cancelling point for this case study was arbitrarily selected as $$ x = 19.8 $$ after some trial and error attempts. This strategy was effective to obtain a low residual error. The resulting system of algebraic equations was solved to obtain the values of $$ A $$ and $$ c $$ and finally an approximate solution for $$ y(x) $$ [see (136)].

**Fig. 2:**
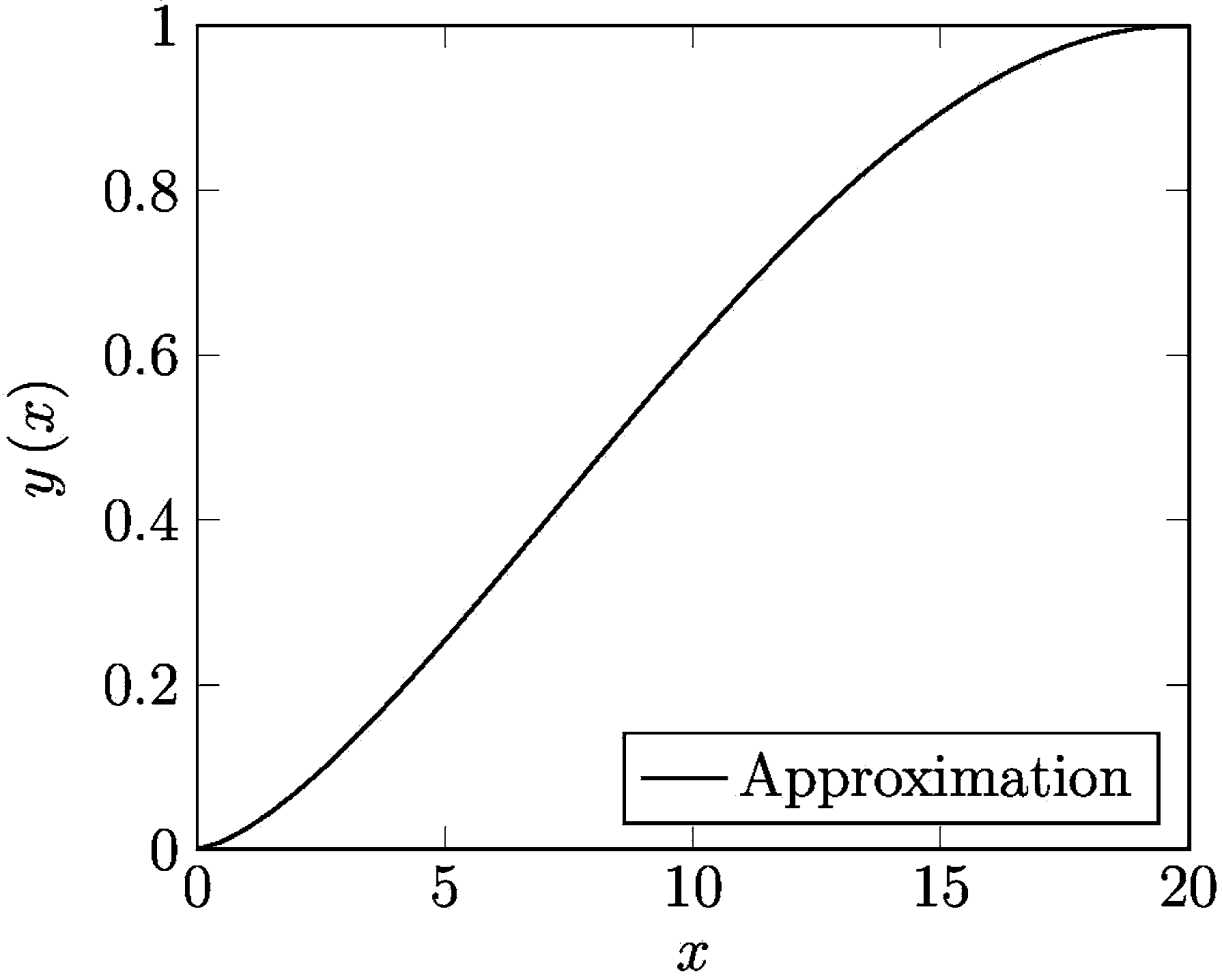
Combination of NDLT-HPM and LT-HPM to obtain approximation (136)

It is worth to note the value of the SRE of (136) is just 0.06334882582; from this we depict the accuracy of our approximate solution (see Fig. 2). If more accuracy has to be required, one must considerer higher order approximations. An outstanding point is that, numerical routines from Maple 15 failed in this case in order to provide a numerical solution as reference. Since the numerical solutions are often the only manner to obtain information from a nonlinear problem, LT-HPM method indeed, is a useful strategy capable of supporting approximate methods, especially for cases like this.

Finally, our case study 4, proposed the problem of extremizing (137), which was also introduced in the most general way. Such as it was said $g(x)$ represents an arbitrary function, although having Taylor series. In the sequel, the mathematical steps (137)–(139) and (141)–(147) were perform without assuming a particular $g(x).$

**Fig. 3:**
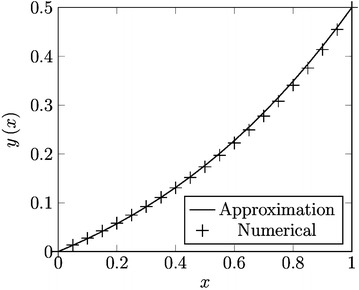
Combination of LT-HPM and NDLT-HPM to obtain approximation (164) (*line*) and numerical solution (*cross*)

**Fig. 4:**
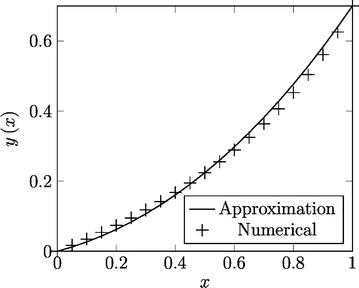
Combination of LT-HPM and NDLT-HPM to obtain approximation (165) (*line*) and numerical solution (*cross*)

We note the proposed homotopy (143) chose the polynomial trial function $$ z(x) = 2Ax $$ and embedded the homotopy parameter $$ p $$ into $$ g(x) $$(see “Basic idea of non-linearities distribution Laplace transform-homotopy perturbation method (NDLT-HPM)” section). (Filobello-Nino et al. 2014b) employed this technique, provided for the freedom of homotopy formulations in order to redistribute the nonlinearities (Vazquez-Leal et al. 2012a) and non-polynomial nonhomogeneous terms, between the successive iterations of LT-HPM method, and thus to ease the search for an approximate solution. The main reason is that NDLT-HPM distributes the contributions of the above mentioned functions along the iterations for the successive parameter powers $$ p $$ that allows to NDLT-HPM method being simpler at the different stages of iterations. As a matter of fact, we proposed a combination between LT-HPM and NDLT-HPM method, in order to obtain better results. As a case study, we considered $$ g(x) = \exp (x) $$ (although for this choosing of $$ g(x) $$ (140) is integrable, it is expected the method works even for cases where (140) cannot be expressed in terms of elementary functions). Its Taylor expansions (152) in terms of the homotopy parameter and further substitution in the proposed approximate solution (150) show the manner which a function is included in the iterative process; in the same way we note the trial function $$ z(x) = 2Ax, $$ was chosen in order to get a whole power series solution, with good precision. With the end of calculating the values of $$ A $$ and $$ c $$ we required that (162) satisfies the boundary condition $$ y(1) = 1/2. $$ Also, following “Basic idea of Laplace transform homotopy perturbation method with variable coefficients (CVLT-HPM)” section, we substituted (162) into (149) and evaluate the expression so obtained for some value, we chose $$ x = 1/5 $$, which lies into $$ 0 \le x \le 1 $$. After performing the above substitution, we obtain a system of algebraic equations for $$ A $$ and $$ c $$, whose solution provided the approximate solution (164). As a matter of fact, proceeding at the same manner, for the proposed second case study given by the conditions $$ y(0) = 0 $$, $$ y(1) = 0.7, $$ we obtained the approximate solution (165). For both cases, we obtained handy accurate approximate solutions; the SRE of (164) and (165) were 0.002934863992 and 0.05009724353 respectively (see Figs. 3, 4), from which we infer that the proposed method is potentially useful in the resolution of the kind of problems as (139). It is possible to obtain more accurate solutions, considering higher order approximations, although could be lost the handy character of our approximation. Worth mentioning even in these case studies, the value of $$ x = 1/5 $$, was chosen by trial and error attempts in order to find a good approximate solution.

It should be mentioned that, variational problems have been successfully studied for other authors, although the most of them have proposed just numerical approximations (Razzaghi and Elnagar 1994; Razzaghi and Yousefi 2001). On the other hand, HAM was employed to obtain an approximate solution for brachistocrone problem (Lakestani and Kazemian 2013). This work obtained precise solutions but long. As it is well known, HAM is accurate and powerful, but sometimes its approximate solutions turn out to be long and cumbersome, and for the same reason, they are not adequate for practical applications; on the other hand we obtained handy accurate approximate solutions with a reduced number of terms. Such as we have already mentioned, unlike (Lakestani and Kazemian 2013) that just studied one variational problem, the goal of this paper was expose in a general manner, how to find analytical solutions for several interesting cases of (2). On the other hand, (Ghaderi 2012) employed homotopy perturbation method (HPM) and Green’s function method in order to find approximate solutions to variational calculus. Nevertheless unlike our work, the mentioned article, dealt with moving boundary and isoperimetric problems. The main advantage of (Ghaderi 2012) is that the mathematical properties of the Green’s function are well established, however its study does not belong to the elementary mathematics; unlike the above, LT-HPM is an iterative method which is based on elementary Laplace transform properties (Spiegel 1998) and simple algebraic steps, making it an ideal technique for practical applications. Finally, future research will be required to improve the present proposal to be applied to infinite intervals.

## Conclusions

This work introduced LT-HPM and some of its modifications in order to find analytical approximate solutions for some Euler’s ordinary differential equations, whose solutions extremizing the value of integrals of the form (1). As it is well known, the calculus of variations is a powerful branch of the analysis that can be applied to several problems of pure mathematics, also to express the basic principles of the mathematical physics in an elegant and simple way.

The relevance of this work consisted mainly on two points. On one hand it showed that the proposed methodology is potentially efficient in order to find solutions to the difficult linear and nonlinear problems arising from variational problems (even CVLT-HPM was able to provide exact solutions, see case study 2). On the other hand the article suggested for some cases, appropriate mathematical manipulations of the aforementioned equations, in order to transform them into a more accessible form, to increase the possibility of success (see cases study 3 and 4). The above is even more important, considering that many times even the numerical methods fail to solve these complicated equations, such as happened with our case study 3, and the numerical solutions and qualitative methods are usually the only manner to obtain information from a nonlinear problem. Finally, an outstanding issue for a future work is improving the methodology used to calculate the parameters introduced for our proposed solutions. A forthcoming paper should investigate a systematic manner to find the optimum values for the aforementioned parameters, without using the method of trial and error attempts in its determination.

## Appendix 1

Laplace transform of $F(t)$ is denoted by $\Im \left\{ {F(t)} \right\}$ and is defined by the integral (Connor and Robertson 1997).

166 $$ \Im \left\{ {F(t)} \right\} = f(s) = \int\limits_{0}^{\infty } {e^{ - st} } F(t)dt. $$

Linearity of Laplace transform is an important property, which is expressed as

167 $$ \Im \left\{ {c_{1} F_{1} (t) + c_{2} F_{2} (T)} \right\} = c_{1} f_{1} (s) + c_{2} f_{2} (s), $$

where $c_{1}$ and $c_{2}$ are constants and we have denoted:$\Im \left\{ {F_{1} (t)} \right\} = f_{1} (s)$, $\Im \left\{ {F_{2} (t)} \right\} = f_{2} (s)$.

Some known properties of LT, widely employed in this study are

1. 168 $$ \Im \left\{ 1 \right\} = \frac{1}{s}\,\,\,\left( {s > 0} \right), $$
2. 169 $$ \Im \left\{ {t^{n} } \right\} = \frac{n!}{{s^{n + 1} }}\,\,\left( {s > 0} \right), $$
3. 170 $$ \Im \left\{ {F^{(n)} (t)} \right\} = s^{n} f(s) - s^{n - 1} F(0) - s^{n - 2} F^{\prime}(0) - \cdots - F^{(n - 1)} (0), $$
  where $F^{(n)} (t)$ denotes the $n$th derivative of $F(t)$ and $\Im \left\{ {F(t)} \right\} = f(s).$
4. 171 $$ \Im \left\{ {t^{n} F(t)} \right\} = ( - 1)^{n} \frac{{d^{n} f(s)}}{{dt^{n} }},\,\,n\,\hbox{denotes a positive integer}, $$
  where $$ \Im \left\{ {F(t)} \right\} = f(s). $$

If Laplace transform of $F(t)$ is $f(s)$, then $F(t)$ is called the inverse Laplace transform of $f(s)$ and is expressed by $F(t) = \Im^{ - 1} \left\{ {f(s)} \right\},$ where $\Im^{ - 1}$ is called the inverse Laplace transform operator.

From Eqs. (168) and (169) is clear that

172 $$ 1 = \Im^{ - 1} \left( {\frac{1}{s}} \right), $$

173 $$ t^{n} = \Im^{ - 1} \left( {\frac{n!}{{s^{n + 1} }}} \right), $$

and so on.

The following important result is obtained from (167) and denotes the linearity property of $\Im^{ - 1}$

174 $$ \Im^{ - 1} \left\{ {c_{1} f_{1} (s) + c_{2} f_{2} (s)} \right\} = c_{1} F_{1} (t) + c_{2} F_{2} (t). $$

## Appendix 2

The square residual error (SRE) of an approximate solution is given by

175 $$ \int\limits_{a}^{b} {R^{2} } \left( {u(r)} \right)dr, $$

*a* and *b* are the end points, the residual $R(u(r))$ is defined as

176 $$ R\left( x \right) = L\left( {u(x)} \right) + N\left( {u(x)} \right) - f(x), $$

where $u(r)$ is an approximate solution for the equation to be solved, which generally may depend of some parameters (See the end of “LT-HPM and some of its modified versions” section).

The square residual error is in general terms a positive number, which is representative of the total error committed, by using the approximate solution $u(r)$ SRE, would be zero only for the case where $u(r)$ turns out to be the exact solution of the differential equation under study.
